# Environmental Humidity Regulates Effects of Experimental Warming on Vegetation Index and Biomass Production in an Alpine Meadow of the Northern Tibet

**DOI:** 10.1371/journal.pone.0165643

**Published:** 2016-10-31

**Authors:** Gang Fu, Zhen Xi Shen

**Affiliations:** Lhasa Plateau Ecosystem Research Station, Key Laboratory of Ecosystem Network Observation and Modeling, Institute of Geographic Sciences and Natural Resources Research, Chinese Academy of Sciences, Beijing, 100101, China; Tennessee State University, UNITED STATES

## Abstract

Uncertainty about responses of vegetation index, aboveground biomass (AGB) and gross primary production (GPP) limits our ability to predict how climatic warming will influence plant growth in alpine regions. A field warming experiment was conducted in an alpine meadow at a low (4313 m), mid- (4513 m) and high elevation (4693 m) in the Northern Tibet since May 2010. Growing season vapor pressure deficit (VPD), soil temperature (*T*_*s*_) and air temperature (*T*_*a*_) decreased with increasing elevation, while growing season precipitation, soil moisture (SM), normalized difference vegetation index (NDVI), soil adjusted vegetation index (SAVI), AGB and GPP increased with increasing elevation. The growing season *T*_*a*_, *T*_*s*_ and VPD in 2015 was greater than that in 2014, while the growing season precipitation, SM, NDVI, SAVI, AGB and GPP in 2015 was lower than that in 2014, respectively. Compared to the mean air temperature and precipitation during the growing season in 1963–2015, it was a warmer and wetter year in 2014 and a warmer and drier year in 2015. Experimental warming increased growing season *T*_*s*_, *T*_*a*_,VPD, but decreased growing season SM in 2014–2015 at all the three elevations. Experimental warming only reduced growing season NDVI, SAVI, AGB and GPP at the low elevation in 2015. Growing season NDVI, SAVI, AGB and GPP increased with increasing SM and precipitation, but decreased with increasing VPD, indicating vegetation index and biomass production increased with environmental humidity. The VPD explained more variation of growing season NDVI, SAVI, AGB and GPP compared to *T*_*s*_, *T*_*a*_ and SM at all the three elevations. Therefore, environmental humidity regulated the effect of experimental warming on vegetation index and biomass production in alpine meadows on the Tibetan Plateau.

## Introduction

By the end of this century, the global surface temperature will increase by 1.0–3.7°C and the alpine ecosystems will be most likely sensitive to such climatic warming in the high altitudes and latitudes regions [1–3]. Low temperature is a key limiting factor in alpine regions, indicating that climatic warming can generally stimulate alpine plant growth and biomass accumulation. For example, A meta-analysis showed that experimental warming increased aboveground plant production in alpine regions [3]. Liu et al. [4], Qin et al. [5], Wang et al. [6], and Zhang et al. [7] found that experimental warming increased plant production in the alpine meadow on the Tibetan Plateau. Considering that experimental warming can increase evapotranspiration and result in drying [8,9], the positive effect of experimental warming on alpine plant growth may be dampened or masked by experimetal-warming-induced environmental (soil and air) drying. For example, experimental warming did not affect plant biomass in alpine meadows on the Tibetan Plateau [10–12]. Experimental warming even decreased gross primary production in an alpine meadow near the Nam Tso Monitoring and Research Station [13]. Therefore, there remain uncertainties about how climatic warming will affect alpine plant growth and associated vegetation index.

Vegetation indices (e.g. normalized difference vegetation index, NDVI; green NDVI, GNDVI; and soil adjusted vegetation index, SAVI), as important indicators of plant growth conditions, are related to plant biomass, primary production and phenology [14–19]. Understanding the responses of these vegetation indices to climatic change is very important for predicting future changes in the plant growth. In order to investigate how climatic change will affect vegetation index, many studies have analyzed the relationships between various vegetation indices (e.g. NDVI) and climatic factors (e.g. air temperature and precipitation) [20,21]. However, the vegetation indices are derived from remote sensing data for most of these studies [20,21]. The satellite-based vegetation indices are not only affected by climatic change, but also influenced by human activities (e.g. grazing and fertilization) [21]. The relationships between the remote-sensing-based vegetation indices and climatic factors may be dampened or strengthed by human actitives. Field warming experiments can reduce and even exclude the disturbance of human activities on the vegetation indices. Few studies have analyzed the response of ground-based vegetation indices to warming at field experimental site scale [14,22,23]. Therefore, it remains unclear how climatic warming will affect vegetation index and more field warming experiments should focus on this issue.

Gross primary production (GPP) or aboveground plant biomass (AGB) are important carbon pools [24]. Compared to vegetation indices, more studies have examined the responses of GPP and/or AGB to experimental warming [3,25]. Their responses to climatic warming vary with vegetation types and climatic conditions at the global scale [24] and on the Tibetan Plateau [2]. Experimental warming increases aboveground plant carbon when warming duration is <5 years, but does not affect aboveground plant carbon when warming duration is within a range from 5 to 10 years at the global scale [24]. Experimental warming may have a lag effect on aboveground plant biomass on the Tibetan Plateau [26]. The warming duration of warming experiments, which focus in the effects of experimental warming on alpine plant growth, is generally <5 years on the Tibetan Plateau. These findings imply that it is necessary to examine the response of alpine plant production to long-term (> 5 years) experimental warming on the Tibetan Plateau. Some studies indicated that the response directions of plant biomass and primary production to experimental warming varied with years [27–29], while other studies demonstrated that their response directions did not change with years [6,30] on the Tibetan Plateau. Therefore, it is unclear whether the responses of biomass production to climatic warming vary with years or whether the responses of biomass production to short-term (<5 years) experimental warming is different from those to long-term (> 5 years) experimental warming on the Tibetan Plateau.

In this study, a field warming experiment was conducted in 2010 at three alpine meadow sites of the Northern Tibet. The monthly NDVI, GNDVI, SAVI, GPP and AGB in 2014 (the fifth year) and 2015 (the sixth year) were measured. The main objective of this study was to examine whether or not experimental warming had a variable effect on biomass production by comparing the response of plant characteristics (i.e. vegetation index, AGB and GPP) between years and the related probable causes in an alpine meadow in the northern Tibet.

## Materials and Methods

### Study Area and Experimental Design

**Fig 1:**
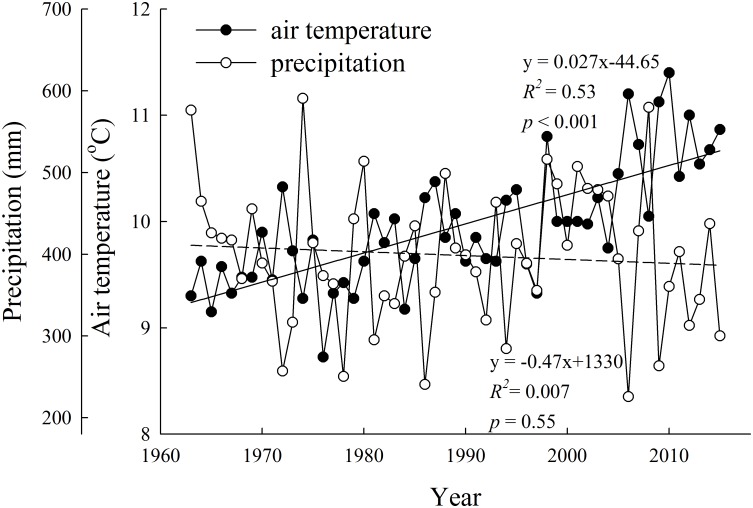
Variation of annual average air temperature and precipitation during the growing season from 1963–2015 in Damxung County meterological station.

The study area (30°30′–30°32′ N, 91°03′–91°04′ E) was located at the Damxung Grassland Observation Station, Tibet Autonomous Region of China. Annual mean air temperature and mean precipitation is 1.9°C and 474.9 mm, respectively. Mean air temperature and mean precipitation during the growing season is 10°C and 398.7 mm, respectively (Fig 1). Mean air temperature during the growing season increases at a rate of 0.027°C y^-1^, while mean precipitation during the growing season shows a non-significant decreasing trend (Fig 1), indicating that growing season becomes warming and drying in this study area. Soil texture is sandy loam and soil layer is 0.5–0.7 m thick. Soil organic matter and total nitrogen is 0.3–11.2% and 0.03–0.49%, respectively. The typical vegetation type in the study area is *Kobresia*-dominated alpine meadow. Roots are mainly concentrated in the topsoil layer (0–20 cm). Both the Damxung Grassland Observation Station and the village leaders gave us permission to conduct our study on their land.

Open top chambers (OTCs), which were used to increase temperature, were established on a south-facing slope on the Nyainqentanglha Mountains at a low (4313 m) mid- (4513 m) and high (4693 m) elevation in May 2010. OTCs has been widely used in field warming experiments in various alpine ecosystems on the Tibetan Plateau [4,9,11,12,28,31–40]. There were four paired OTCs and control plots at each site. There was approximately 3.00 m distances between plots. The OTCs were 0.40 m, 1.45 m and 1.00 m in height, bottom and top diameters. Air temperature decreased with increasing elevation and precipitation increased with increasing elevation along the elevation gradient [41]. The dominant species were *Stipa capillacea*, *Carex montis-everestii* and *Kobresia pygmaea* at the low elevation, *Stipa capillacea*, *Kobresia pygmaea* and *Carex montis-everestii*, at the mid-elevation, and *Kobresia pygmaea* at the high elevation [41]. The importance of *Kobresia pygmaea* increased with increasing elevation [42].

### Microclimate Observations

At each elevation, meteorological stations (HOBO weather station, Onset Computer, Bourne, MA, USA) were used to monitor soil temperature (*T*_*s*_) at a depth of 0.05 m, soil moisture at a depth of 0.10 m (SM), air temperature (*T*_*a*_) and relative humidity (RH) at a height of 0.15 m during the growing season in 2014–2015. Measured *T*_*a*_ and RH was used to calculate vapor pressure deficit (VPD). Mean growing season precipitation (MGSP) at the three elevations were obtained from our previous study [41].

### Vegetation Index

A Tetracam Agricultural Digital Camera (ADC, Tetracam Inc., Chatsworth, CA, USA) was ued to obtain photographs in a 0.50 m × 0.50 m subplot in the center of each plot during the growing season (June-September) in 2014–2015. Calibration photographs of a white teflon plate provided by the Tetracam manufacturer were taken before and after each batch of the study target photographs, and also taken when light condition changed remarkably. The PixelWrench2 software were used to obtain normalized difference vegetation index (NDVI), green normalized difference vegetation index (GNDVI) and soil adjusted vegetation index (SAVI) [43,44].

### GPP Algorithm

We calculated GPP based on the Moderate Resolution Imaging Spectroradiometer (MODIS) GPP algorithm. The detailed description on the MODIS algorithm can be found in our previous study [45].

GPP=PAR×FPAR×LUE (1)

$$LUE=LUE_{\text{max}}\times T_{\text{a min}}{\_}_{scalar}\times VPD{\_}_{scalar}$$ (2)

$$T_{\text{a min}}{\_}_{scalar}=\{\begin{array}{cc} 0, & T_{\text{a min}}<T_{\text{a min}}{\_}_{\text{min}}\\ \frac{T_{\text{a min}}-T_{\text{a min}}{\_}_{\text{min}}}{T_{\text{a min}}{\_}_{\text{max}}-T_{\text{a min}}{\_}_{\text{min}}}, & T_{\text{a min}}{\_}_{\text{min}}<T_{\text{a min}}<T_{\text{a min}}{\_}_{\text{max}}\\ 1, & T_{\text{a min}}>T_{\text{a min}}{\_}_{\text{max}} \end{array}$$ (3)

$$VPD{\_}_{scalar}=\{\begin{array}{cc} 0,\; & VPD>VPD_{\text{max}}\\ \frac{VPD_{\text{max}}-VPD}{VPD_{\text{max}}-VPD_{\text{min}}}, & VPD_{\text{min}}<VPD<VPD_{\text{max}}\\ 1, & VPD<VPD_{\text{min}} \end{array}$$ (4)

where PAR is photosynthesis active radiation; LUE_max_ is the maximum light use efficiency (g C MJ^-1^), and was 0.81 g C MJ^-1^ in the current study [45]; FPAR, i.e., the fraction of absorbed PAR by vegetation canopy. FPAR was estimated using the observed NDVI values in this study [46]

$$FPAR\;=\frac{FPAR_{NDVI}+FPAR_{SR}}{2}$$ (5)

$$FPAR_{NDVI}=\frac{\left(NDVI-NDVI_{\text{min}})(FPAR_{\text{max}}-FPAR_{\text{min}}\right)}{NDVI_{\text{max}}-NDVI_{\text{min}}}+FPAR_{\text{min}}$$ (6)

$$FPAR_{SR}=\frac{\left(SR-SR_{\text{min}})(FPAR_{\text{max}}-FPAR_{\text{min}}\right)}{SR_{\text{max}}-SR_{\text{min}}}+FPAR_{\text{min}}$$ (7)

$$SR\;=\frac{1+NDVI}{1-NDVI}$$ (8)

where *FPAR*_*max*_ = 0.95 and *FPAR*_*min*_ = 0.001, respectively [46].

### AGB Estimation

AGB was estimated using a non-destructive approach which was based on a regression equation (AGB = 10.33exp^3.28NDVI^, *R*^*2*^ = 0.64, *p* < 0.001, *n* = 135) [47].

### Statistical Analysis

For each site, a *t*-test was used to estimate the effect of experimental warming on growing season average *T*_s_, *T*_*a*_, SM and VPD. Repeated-measures analysis of variance was used to estimate the main and interactive effects of experimental warming and sampling months on NDVI, GNDVI, SAVI, GPP and AGB. Single factor regression analysis between NVDI, GNDVI, SAVI, GPP, AGB and SM, VPD and MGSP were conduced, respectively. Multiple stepwise linear regression analyses between NVDI, GNDVI, SAVI, GPP, AGB and *T*_s_, *T*_*a*_, SM and VPD were conduced, respectively. All the statistical analyses were performed using the SPSS software (version 16.0; SPSS Inc., Chicago, IL).

## Results and Discussion

At the low elevation, the average *T*_*s*_, *T*_*a*_, and VPD in 2015 was 0.82°C, 0.89°C and 0.30 kPa greater than that in 2014, respectively (*p* < 0.05), regardless of experimental warming, while the average SM, NDVI, SAVI, AGB and GPP in 2015 was 0.06 m^3^ m^-3^, 0.10, 0.08, 8.45 g m^-2^ and 0.41 g C m^-2^ d^-1^ lower than that in 2014, respectively (*p* < 0.05). At the mid-elevation, the average *T*_*s*_, *T*_*a*_, and VPD in 2015 was 0.83°C, 1.10°C and 0.25 kPa greater than that in 2014, respectively (*p* < 0.05), regardless of experimental warming, while the average SM, NDVI, SAVI, AGB and GPP in 2015 was 0.05 m^3^ m^-3^, 0.15, 0.11, 16.64 g m^-2^ and 0.52 g C m^-2^ d^-1^ lower than that in 2014, respectively (*p* < 0.05). At the high elevation, the average *T*_*s*_ and VPD in 2015 was 0.28°C and 0.15 kPa greater than that in 2014 (*p* < 0.05), regardless of experimental warming, while the average NDVI, SAVI, AGB and GPP in 2015 was 0.22, 0.18, 35.57 g m^-2^ and 1.02 g C m^-2^ d^-1^ lower than that in 2014, respectively (*p* < 0.05). The air temperature in 2014 and 2015 was 10.67°C and 10.86°C, and the precipitation was 437.3 mm and 300.2 mm, respectively, indicating that the precipitation in 2014 and 2015 were greater and lower than the mean precipitation of 1963–2015 (i.e. 398.7 mm) (Fig 1). These results implied that it was a warmer and drier year in 2015 with lower vegetation index, AGB and GPP than in 2014. That is, the interannual variations of AGB, GPP and vegetation index were in line with those of precipitation and SM, but in contrast with those of VPD, *T*_*s*_ and *T*_*a*_. Likewise, annual gross primary production was twice in a wet year than in a dry year in a semi-arid grassland in Hungary [48]. The significant inter-annual variations of vegetation index, AGB and GPP were also in line with previous studies conducted on the Tibetan Plateau [26,27].

Regardless of experimental warming and measuring year, *T*_*s*_, *T*_*a*_ and VPD declined with increasing elevation, while SM, NDVI, GNDVI, SAVI, AGB and GPP increased with increasing elevation (*p* < 0.001). The MGSP increased with increasing elevation [41]. These indicated that the vegetation index, AGB and GPP showed consistent trends with SM and precipitation, but showed quite the contrary trends with *T*_*s*_, *T*_*a*_, and VPD along the elevation gradient. Similarly, Wang et al. [42] found that precipitation, aboveground and belowground plant biomass increased with increasing elevation, but air temperature decreased with increasing elevation from 4400 m to 4800 m in an alpine meadow of the Northern Tibet. The AGB and mean annual precipitation in our study site was lower than that in the Haibei station, whereas mean annual air temperature was greater [6,30,49]. These findings implied that the alpine meadow site with a warmer and drier climatic condition may have a lower vegetation index and biomass production.

**Fig 2:**
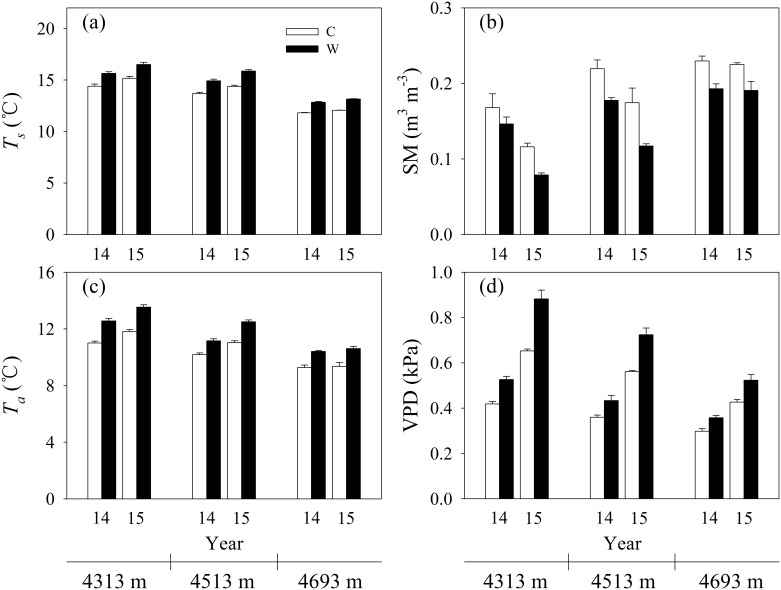
Effects of experimental warming on soil temperature (*T*_*s*_), soil moisture (SM), air temperature (*T*_*a*_), and vapor pressure deficit (VPD) at elevation 4313 m, 4513 m, and 4693 m in 2014 and 2015 in an alpine meadow of the Northern Tibet. Error bars represent standard errors (*n* = 4).

**Fig 3:**
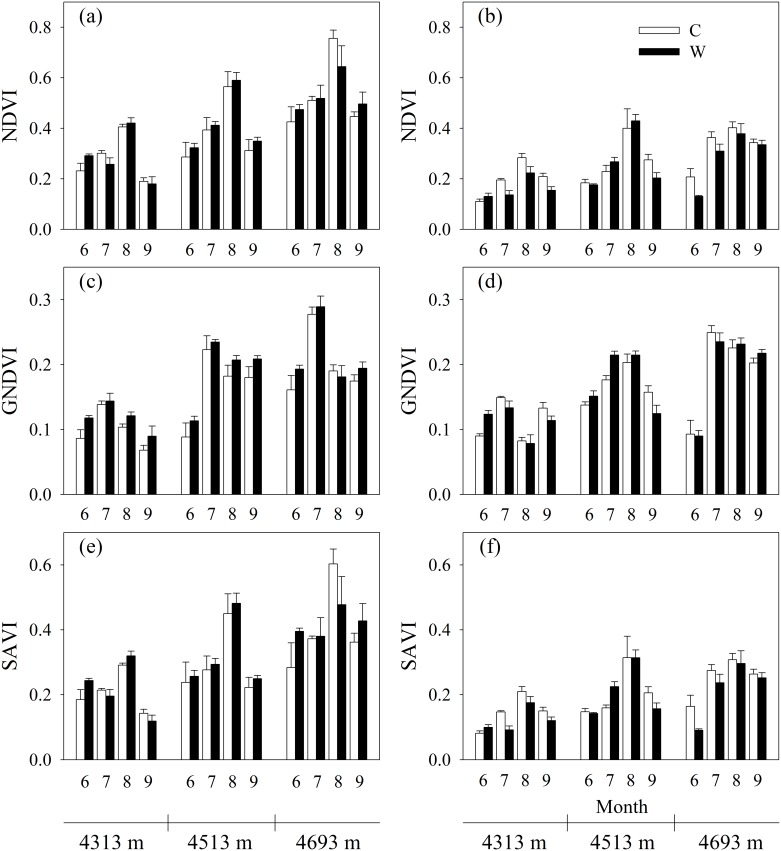
Effects of experimental warming on normalized difference vegetation index (NDVI), green NDVI (GNDVI), and soil adjusted vegetation index (SAVI) at elevation 4313 m, 4513 m, and 4693 m in 2014 and 2015 in an alpine meadow of the Northern Tibet. Error bars represent standard errors (*n* = 4).

**Fig 4:**
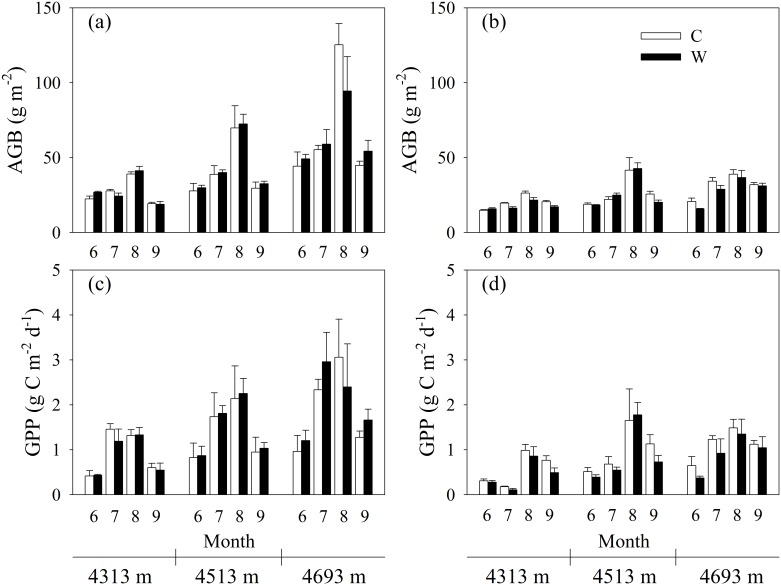
Effects of experimental warming on aboveground plant biomass (AGB, g m^-2^) and gross primary production (GPP, g C m^-2^ d^-1^) at elevation 4313 m, 4513 m, and 4693 m in 2014 and 2015 in an alpine meadow of the Northern Tibet. Error bars represent standard errors (*n* = 4).

Compared to the control, experimental warming increased average *T*_*s*_ by 1.24, 1.23 and 1.04°C in 2014, and 1.33, 1.49 and 1.09°C in 2015 at the low, mid- and high elevation, respectively (Fig 2). Experimental warming increased average *T*_*a*_ by 1.55, 0.98 and 1.13°C in 2014, and 1.73, 1.49 and 1.28°C in 2015 at the low, mid- and high elevation, respectively (Fig 2). In contrast, experimental warming decreased average SM by 12.9%, 19.1%, 16.0% in 2014, and 32.0%, 32.8% and 15.2% in 2015 at the low, mid- and high elevation, respectively (Fig 2). Experimental warming increased average VPD by 0.11, 0.07 and 0.06 kPa in 2014, and 0.23, 0.16 and 0.10 kPa in 2015 at the low, mid- and high elevation, respectively (Fig 2). These results implied that experimental warming resulted in a warming and drying environmental condition, which was in line with the climatic change trends in Damxung County meterological station during the past 53 years (Fig 1). Experimental warming-induced drying may be mainly due to the increase in evapotranspiration caused by experimental warming [8]. The climate became warming and drying across the alpine meadow meteorological stations on the Tibetan Plateau during the past 13 years [21]. The warming and drying climatic change resulted in 3.9% decline in growing season maximum normalized differnce vegetation index over the alpine meadow meteorological stations on the Tibetan Plateau [50]. At the low elevation, experimental warming reduced average NDVI by 19.5%, SAVI by 17.0% (Fig 3), AGB by 12.8% (-2.59 g m^-2^) and GPP by 22.4% (-0.13 g C m^-2^ d^-1^) (Fig 4) in 2015. The minimum SM for meadow growth was 11.8% [51]. The average SM in 2015 was lower than 11.8% under the control plots and experimental warming reduced SM at the low elevation (Fig 2), which may result in reductions in biomass production. However, experimental warming did not affect vegetation index, AGB and GPP in 2014 which was a relative colder and wetter year compared to the year of 2015 (Figs 3 and 4). Experimental warming also reduced average GPP and AGB in 2012 in the alpine meadow at the low elevation [52] and it was also a drier year of 2012 (312.8 mm) compared to the mean precipitation of 1963–2015 (Fig 1). These implied that the response directions of plant growth to experimental warming varied with years and experimental warming did not have a continuous negative effect on biomass production at the low elevation, which was in line with previous studies [27–29,53,54]. The variable response directions may be due to the variable environmental temperature and humidity among years [27,29] and plant growth was more sensitive to moisture than temperature change when water was lower than a certain threshold value [53,55]. The precipitation in 2014 was 38.6 mm higher compared to the mean precipitation (398.7 mm), whereas the precipitation in 2012 and 2015 was 85.88 mm and 98.48 mm lower compared to the mean precipitation, respectively. These implied that experimental warming likely had a negative effect on plant growth when the precipitation was lower a certain threshold value. However, our finding was an advancement over some previous studies conducted on the Tibetan Plateau, which showed that experimental warming continuously increased or decreased biomass production. For example, Klein et al. [30] indicated that experimental warming continuously reduced aboveground net primary production in an alpine meadow in the Haibei station in 1999–2001. Lin et al. [49] found that experimental warming continuously increased aboveground and belowground plant biomass in an alpine meadow in the Haibei station in 2006–2008. Wang et al. [6] demonstrated that experimental warming continuously increased aboveground net primary production in an alpine meadow in the Haibei station in 2006–2010. Moreover, the precipitation changes should be considered when we compare the effects of short-term and long-term experimental warming on aboveground biomass and gross primary production.

**Fig 5:**
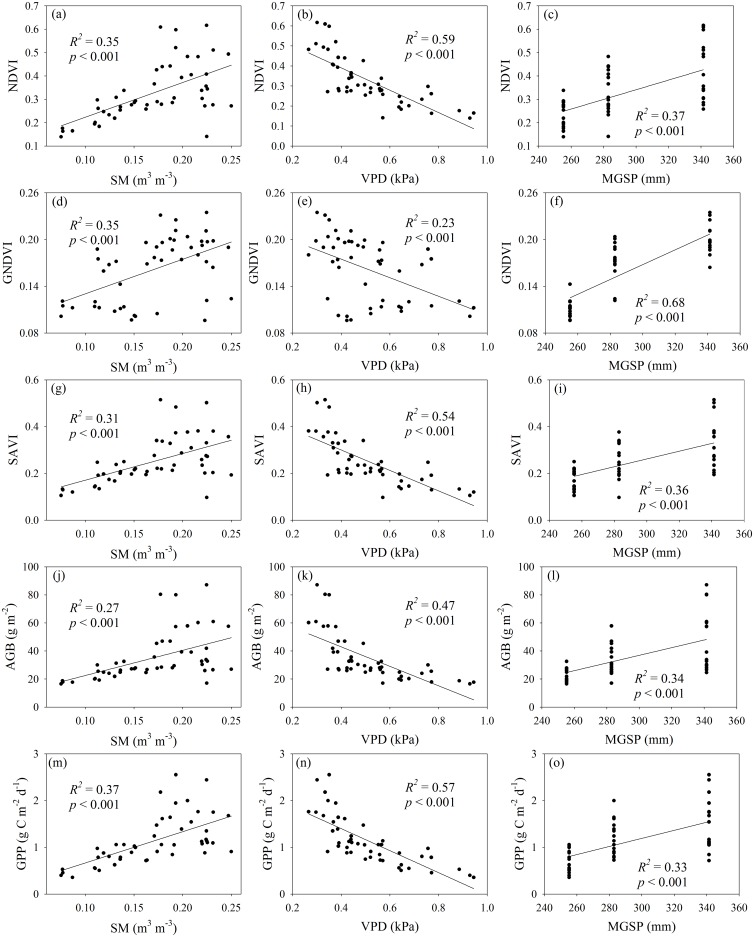
Relationships of average normalized difference vegtation index (NDVI), green normalized difference vegtation index (GNDVI), soil adjusted vegetation index (SAVI), aboveground plant biomass (AGB, g m^-2^), gross primary production (GPP, g C m^-2^ d^-1^) with soil moisture (ΔSM, m^3^ m^-3^), vapor pressure deficit (VPD, kPa) and mean growing season precipitation (MGSP, mm) across elevation and warming treatment in an alpine meadow on the Tibetan Plateau. MGSP data at the three elevations were obtained from Fu et al. [41].

Regardless of measuring year, experimental warming reduced average NDVI by 7.1% and GPP by 13.1% at the low elevation rather than the mid- and high elevation. This was most likely attributed to their different environmental humidity conditions among the three alpine meadow sites considering that NDVI, GNDVI, SAVI, GPP and AGB showed a positive correlation with MGSP and SM and a negative correlation with VPD (Fig 5). Similarly, a l.8°C increase in soil temperature increased AGB in wet but decreased AGB in dry conditions in an alpine meadow situated in the source region of the Yangtze River and in the middle of the Tibetan Plateau [55]. A recent meta-analysis indicated that the effect of warming on plant biomass increased with increasing mean annual precipitation when mean annual precipitation was lower than 600 mm [56]. Wu et al. indicated that ANPP increased significantly increasing precipitation in alpine grasslands of the northern Tibet [57]. Another meta-analysis indicated that the effect of warming on aboveground plant productivity decreased with annual air temperature [3]. The change of maximum enhanced vegetation index was more sensitive to warming in colder environments across all the meterological stations on the Tibetan Plateau [21]. These findings implied that the response of plant growth to warming was dependent on the local environments.

Although several previous studies found that experimental warming-induced changes in biomass production may be related to warming magnitudes in alpine regions [2,23,28,50,58,59], the change magnitudes in NDVI, GNDVI, SAVI, AGB and GPP caused by experimental warming were not correlated with those in *T*_*s*_, *T*_*a*_, SM and VPD in the current study (S1 Fig). Experimental warming-induced change magnitudes in *T*_*s*_ and *T*_*a*_ in 2014 was not different from those in 2015. There were no differences of experimental warming-induced change magnitudes in *T*_*s*_ and *T*_*a*_ among the three elevations. Therefore, warming magnitudes may not the reason which resulted in the varialbe responses of vegetation index, AGB and GPP to experimental warming among the elevation and between the year.

More variations of NDVI, SAVI, AGB and GPP were explained by VPD at all the three elevations compared to *T*_*s*_, *T*_*a*_ and SM (Table 1), and more variation of GNDVI was explained by SM compared to VPD (Fig 5). These implied that environmental humidity condition significantly affect vegetation index and biomass production in this study. Similarly, Precipitation was the main climatic factor in affecting vegetation index and coverage for the alpine grasslands on the Tibetan Plateau [60]. Precipitation dominated the variations of aboveground and blowground plant biomass along an elevation gradient from 4400 m to 4800 m in an alpine meadow in the Northern Tibet [42]. Soil moisture explained more variation of GPP than soil temperature under experimental warming condition in an alpine meadow in the Naqu county, Tibet [27].

**Table 1:** Multiple stepwise linear regression analyses between normalized difference vegetation index (NDVI), green NDVI (GNDVI), soil adjusted vegetation index (SAVI), gross primary production (GPP), aboveground plant biomass (AGB) and soil temperature (*T*_s_), air temperature (*T*_*a*_), soil moisture (SM) and vapor pressure deficit (VPD), showing the regression coefficient, significance probability (*p*), and coefficient of determination (*R*^*2*^).

Variable		Low elevation			Mid-elevation			High elevation		
		Coefficient	*R*^*2*^	*P*	Coefficient	*R*^*2*^	*P*	Coefficient	*R*^*2*^	*P*
NDVI	Constant	0.01		0.892	1.18		<0.001	1.47		<0.001
	SM				-2.10	0.29	0.003	-2.34	0.14	0.012
	*T*_*a*_	0.04	0.15	0.002						
	VPD	-0.47	0.73	<0.001	-0.93	0.44	<0.001	-1.41	0.65	<0.001
SAVI	Constant	-0.04		0.567	0.93		<0.001	1.26		<0.001
	SM				-1.72	0.30	0.005	-2.22	0.17	0.007
	*T*_*a*_	0.04	0.20	<0.001						
	VPD	-0.37	0.72	<0.001	-0.73	0.37	<0.001	-1.18	0.61	<0.001
AGB	Constant			0.787	122.35		<0.001	229.05		<0.001
	SM				-208.84	0.21	0.015	-419.25	0.15	0.013
	*T*_*a*_	3.66	0.18	<0.01						
	VPD	-38.49	0.68	<0.001	-100.11	0.44	<0.001	-233.18	0.61	<0.001
GPP	Constant	0.23		0.597	2.09		<0.001	-1.74		0.35
	*T*_*s*_							0.48	0.15	0.01
	*T*_*a*_	0.13	0.09	<0.001						
	VPD	-1.70	0.77	0.016	-1.74	0.50	0.002	-6.90	0.65	<0.001

It is well known that nitrogen shortages limit plant growth and primary production in alpine regions [61,62]. Soil microbial activity can affect inorganic nitrogen content [63,64]. Increased temperature and soil drying induced by experimental warming can affect soil enzymes (e.g. urease) and microbial activity [63,65]. Therefore, the effects of experimental warming on soil microbial activity, urease and inorganic nitrogen availability [6,63–65] may also affect alpine vegetation growth and production to some extent.

## Conclusions

The main conclusions are as follows: (1) responses of vegetation index, aboveground plant biomass and gross primary production to warming vary with year and the local environments; and (2) environmental humidity was the main factor affecting the variation of vegetation index and biomass production in the alpine meadow of the Northern Tibet.

## Supporting Information

S1 Fig Relationships between experimental warming-induced changes in average normalized difference vegtation index (ΔNDVI), green normalized difference vegtation index (ΔGNDVI), soil adjusted vegetation index (ΔSAVI), aboveground plant biomass (ΔAGB, g m^-2^), gross primary production (ΔGPP, g C m^-2^ d^-1^), and soil temperature (Δ*T*_*s*_, °C), soil moisture (ΔSM, m^3^ m^-3^), air temperature (Δ*T*_*a*_, °C) and vapor pressure deficit (ΔVPD, kPa) across elevation and warming treatment in an alpine meadow on the Tibetan Plateau. (TIF)
