# Supplementary figures and images for: Environmental Humidity Regulates Effects of Experimental Warming on Vegetation Index and Biomass Production in an Alpine Meadow of the Northern Tibet
Source: PLoS One. 2016 Oct 31;11(10):e0165643. doi: 10.1371/journal.pone.0165643 (PMC5087907; doi:10.1371/journal.pone.0165643)

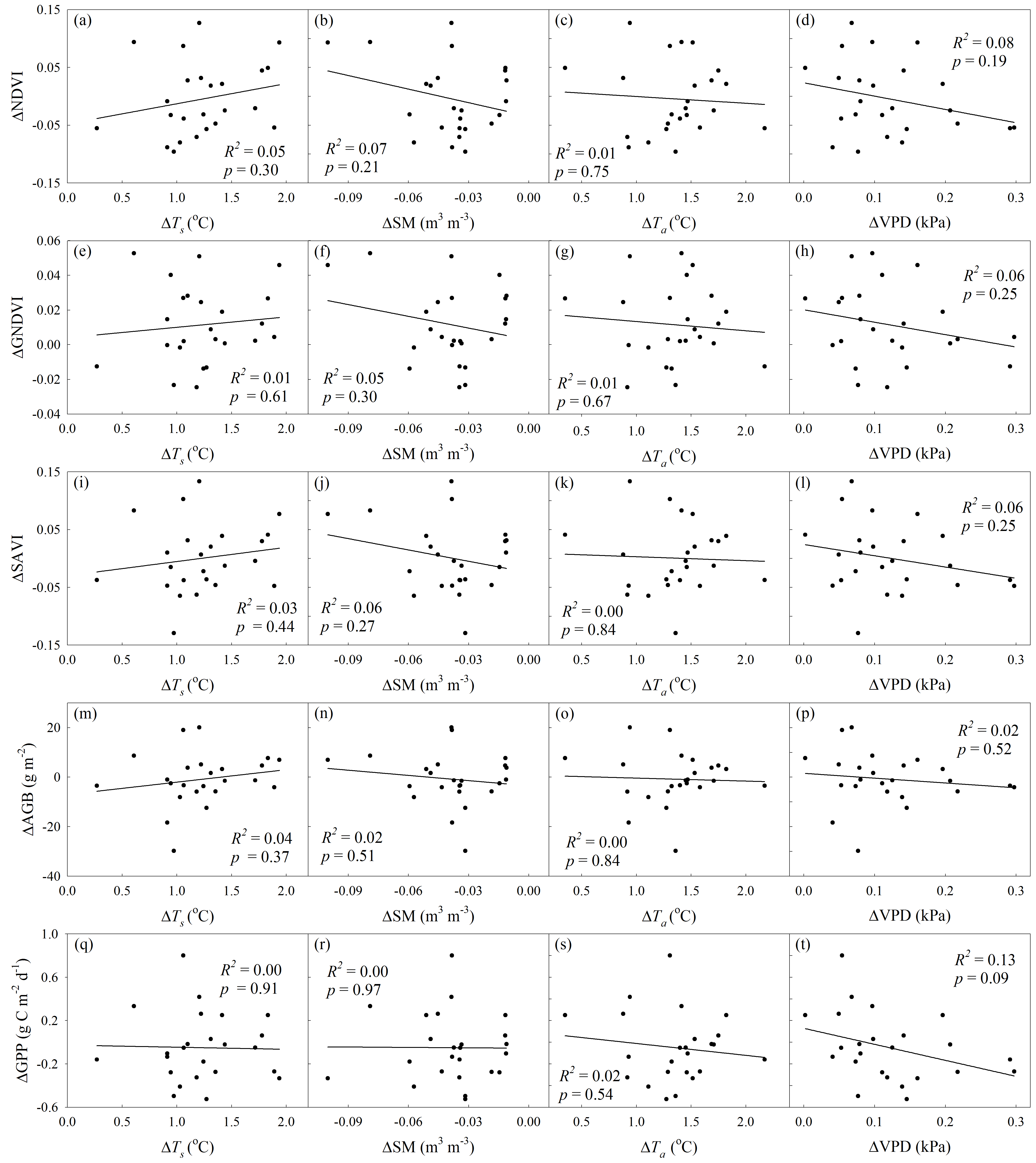

Supplement: S1 Fig — (TIF) [file pone.0165643.s001.TIF]
